# Labour politics as public health: how the politics of industrial relations and workplace regulation affect health

**DOI:** 10.1093/eurpub/cky163

**Published:** 2018-11-01

**Authors:** Scott L Greer

**Affiliations:** Department of Health Management and Policy, University of Michigan, Ann Arbor, MI, USA

## Abstract

There are three main areas of social and economic policy that influence health: the welfare state, industrial organization (unions), and labor regulation. Public health literature and analysis traditionally focuses on the taxing and spending of the welfare state. This paper presents highlights from the extensive literature in political economy in order to argue that industrial relations and workplace regulation are political and crucial to public health. The routes by which they influence public health include wage inequality, workplace health and safety, political engagement and investment in human capital. The magnitude of impact can be impressive: the United Kingdom’s taxation and spending have about as much redistributive impact as that of Sweden, but that is not enough to compensate for the inequality produced by the UK’s liberal labor market. The trend across wealthy countries has been to weaker unions and less workplace regulation and we can see this as a likely cause of public health problems and health inequalities into the future.

Few who are familiar with public health scholarship will dispute the benefits to individual and public health of a strong economy, high-quality and regular employment, an egalitarian and generous welfare state and an egalitarian economic system. The volume of evidence for the benefits of such a political economy in the public health literature worldwide is impressive, as is the still greater volume of evidence showing the negative health effects of unemployment, inegalitarian economic policies, weak public services, precarity, dangerous workplaces and inequality. Public health rarely produces widely sold trade books, but it is noteworthy that the recent ones we have had, synthesizing the literature undergirding his paragraph, have made these points forcefully.^1^^,^^2^

The ‘policy implications’ section of public health articles is often the weakest, and that is true for the literature on these topics. Much work on health inequalities diverts into local- and individual-level policies.^3^ An article that starts out by identifying very big structural drivers of inequality will often divert into much less ambitious policies. The attractiveness of such a move stems in part from the high political stakes—to recommend what is good for public health is often also to recommend what social democrats and parties of the left support. It also comes, I suggest, from the relative weakness of political economy scholarship in public health—in particular scholarship on the political organization of labour markets.

It is a commonplace of comparative social policy that there are three main ways politics and institutions shape individuals’ economic fates. They are the industrial relations system, which is how workers are organized and wages set; labour market regulation, which governs hiring and firing and the welfare state, which is the system for providing benefits.

Industrial relations and workplace regulation receive relatively little attention in public health, and much of what scholarship there is focuses on industrial health and safety. This article puts forth evidence, largely from comparative political economy scholarship, that the impact of labour market institutions on public health is very large and is an area where political action and policy change has major effects on public health. Labour market institutions, often more than taxes, education, spending and technology, govern the extent of inequality, precarity, workforce participation and unemployment in society. They also affect the propensity of governments, individuals and companies to invest in health and human capital. And they are very much political and influenced by policy. As a result, they should be an important focus for public health research.

## Labour power and labour markets

It is customary in many circles to start with a conceptual economic model of labour markets that presents them as free, in a state of nature, with employers and employees matching with each other and salary determined by the marginal cost and contribution of hiring another worker. In such a model, regulation is often residual and to be distrusted. The model is useful enough as a starting point for academic economic inquiry, but it has always had a strong normative dimension that says such a labour market is desirable. What should be a modeller’s convenience turns into a policy objective in the hands of conservative political parties and institutions they influence such as the European Union (e.g. the European Semester). Thus, e.g. the advice given by the EU and OECD to countries as different as France, Italy, Portugal, Ireland and Germany is oddly similar: liberalize labour markets.^4^

In reality, labour markets can and often are structured in complex ways, and they have quite different distributions of power and effects on the economy that political scientists have long sought to understand and synthesize.^5^ There are, crudely, three types:

### Decentralized liberal

This is the kind of labour market that most approximates the economic model and the policy prescriptions of many economists. In it, the core of the labour market is the contract between the individual and the firm. Employer discretion is very substantial, and the remedy offered to unhappy workers is to leave their job for another one. The main protections in these systems will often be about credentialing, occupational safety and health and anti-discrimination provisions rather than efforts to strengthen all employees’ individual or collective bargaining power. These systems are by no means the free markets of simple economic models, but they do not have strong unions and do have substantial employer discretion. The United States and post-Thatcher United Kingdom epitomize this approach.

### Coordinated

At the other extreme is neocorporatism, or social partnership.^6^ Employers and workers are organized into national-level sectoral associations which bargain on wages, working hours, benefits and other issues across entire sectors if not the whole economy. In this model unions are powerful by definition, since they are key to national bargains. They also tend to have unions which are stronger on the shop floor, strong employment protections and collaborative training programmes for workers. The longstanding epitomes of this kind of economy are Austria, Germany, Belgium and the Scandinavian states, though parts of it have been admired and even implemented in quite different economies such as Ireland.

### Dualist

Finally, a dualist economy is an awkward and generally unstable hybrid of the two types in which some parts of the economy, typically the public sector and key industries, have neocorporatist bargaining while much of the rest of the economy, especially the service sector, has a high degree of employer discretion and employee precarity.

There are various routes to this kind of system. Southern European and Latin American economies often have substantial dualism as a legacy of authoritarian regimes which bought off formal-sector workers with lavish union schemes as a mechanism of political control.^7^

In others, it develops as a way-station from neocorporatism to liberalism as unions start to lose power. That appears to be happening in Germany right now, where the effective reach of coordinated bargaining is shrinking and the scope of employer discretion in most sectors increasing.^8^ It is not clear that such dualistic labour markets are actually stable, as against a way-station from coordination to liberalism.^9^

## The impact of different labour markets on public health

The extent of coordination in labour markets is a major driver of public health outcomes. Above all, it contributes directly to equality in beneficial ways.

### Coordinated bargaining and wage compression

The most important impact of labour politics on public health comes through the impact of different types of labour market coordination on inequality in society. In coordinated labour markets, wages are substantially set by national peak-level agreements. In these negotiations, economy-wide union federations generally fight for ‘wage compression’, which means they fight to diminish the gap between the best and the worst paid employees. This reduces the gap between the most and least rewarded employees that is often seen in more flexible labour markets. The result has been a longstanding correlation between higher levels of stable, coordinated, bargaining and lower inequality.^10–12^

Public health research often uses an implicit model of the political economy that focuses on taxes and spending as the main policy tools and fails to develop much understanding of the labour markets that are the key distributors of work, income, wealth and value in society. As a result, we miss a key driver of inequality in wages, life chances and health—the UK government is about as redistributive as the Swedish, but the very unequal ‘predistributive’ effects of its liberal labour market on income give the UK a much more unequal outcome than Sweden even after taxes and spending. Labour market coordination does not address inequalities in wealth and investment income that explain the disproportionate wealth of the top 1% globally,^13^ but it might increase the likelihood of a politics that does.

### Shopfloor worker power and occupational health and safety

Coordinated labour markets with strong unions also limit employer discretion on the shopfloor. This can irritate managers and reproduce a wide variety of inequalities, but it can have health and safety benefits since it gives workers powerful institutional resources with which to speak out against dangerous or unhealthy workplaces and work practices. Organized shopfloor labour is a useful buttress—and flexible alternative—to regulation.

### Incentive to invest in human capital

Unions protect workers’ job security whenever possible, whether in negotiations or in legislative pressure. The result is that strong unions generally correlate with more protection for employees. This, in turn, means that firms have incentives to invest in the skills and health of their employees since it is harder to get rid of them should their skills become obsolescent or their health fail. Part of the manufacturing miracle associated with Germany, and nearby countries such as Austria, lies in a system in which firms with highly protected workers invest in their skills.^14^ More stable employment is not just a health benefit in itself. It creates incentives for employers to invest in training and workplace flexibility that has social and economic benefits.^15^

### Wellness, occupational safety and health

In much of Europe the public health system separates employers from the direct financial costs of health care for employees. This attenuates the direct relationship between employee health and the company’s bottom line, though many employers do provide some amount of health care to their workers (e.g. private health care insurance) and therefore have some exposure to workers’ health. The impact of ill health on worker productivity, nonetheless, matters to companies which are dependent on a high-skilled workforce and creates incentives to greater workplace health and safety initiatives than would be found in economies with more flexible labour markets and less employer dependence on skilled employees (early retirement has been a common way for employers to rid themselves of staff in such economies, but it is not cheap). Unsafe and unhealthy workplaces are less attractive options for employers if the employees are not easily dismissed and replaced.^16^

### ‘Presenteeism’ and public health

It is not an accident that the word ‘presenteeism’ was coined in the USA, which has notably weak unions and workplace regulation. It refers to the presence of sick employees at work. The topic has mostly been discussed in the context of the losses to employers when employees come to work sick, but it obviously has also effects on public health if employees come to work ill and infect colleagues or the public. In some areas such as food service and health care presenteeism is therefore a clear public health challenge. The solution is of course sick days and labour arrangements that do not punish employees for using them. Such a policy depends on either particular labour regulation (e.g. mandatory sick days) or strong union power.

### Gender and racial equality

With regards to gender, racial and other equalities, the track record of all three kinds of labour market is less clear. Unions have often had a strong masculine bias and were an integral part of welfare systems built around male breadwinners. They also often had a role in closing occupations or whole parts of the economy to nonwhites, women or ethnic minorities. This partly accounts for the relatively high employment rates for those groups in decentralized liberal economies. In some coordinated labour markets, however, unions and employers have seen a shared interest in greater equality. Thus, e.g. Sweden’s performance in gender equality is in large part due to wage compression. Likewise, unions have found in many recent cases that supporting immigrant and minority integration is better than excluding them and creating reserves of labour that might undercut union workers. Dualist systems, which in Europe tend to have organized manufacturing and public sector, might have the worst prospects since they focus labour market policy on manufacturing sectors and increasingly disregard the interests of the often more female service sectors, or set up distributive conflict between public and private as a whole.

### Class biases in political engagement

Finally, unions are effective devices for shaping both voters’ political consciousness and the character of political elites.^17^ Strong unions counteract the tendency of politics to become the preserve of the educated upper classes while contributing to the everyday political education and engagement of workers. In the absence of strong unions—an absence increasingly common across Europe—the result is a less organized, more media-dominated politics with fewer leaders from the working class and shallower social roots.^18^ It is hard not to suspect that such a politics leads to the declining political trust and populist politics we see in many countries today.

## Conclusions

**Figure 1:**
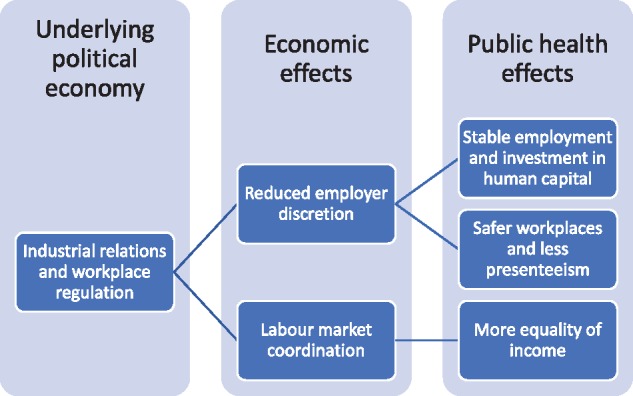
The argument in summary

One of the implications of the analysis above, is that unions are still by far the best advocates for a healthy political economy and are also among the best mechanisms for the implementation of healthy labour markets. Unions are not the object of much public health scholarship and are often negatively viewed. Elements of organized labour can be critiqued, from its putative contribution to dualistic labour markets (and therefore precarity) in some countries to frustrating work rules, but the costs to public health of union decline are considerable. It is very likely that the disappointing public health performance of the USA, in particular, can be related to its low and declining unionization through the vectors in figure 1.

In the context of the Eurozone, no country has been able to thrive without a coordinated labour market.^19^^,^^20^ This finding stands in complete opposition to the focus of EU policies on liberalization.

In the absence of unions strong enough to maintain some labour market centralization and shopfloor worker power, there is more pressure on the state to promote public health through economic, labour and regulatory policy—in other words, compensate for the absence of neocorporatist mechanisms that once led private actors to produce public goods. This is sometimes called the ‘flexicurity’ approach and attributed to Denmark, where it is at best a partial explanation of Danish performance given Denmark’s highly coordinated labour markets. Really, it was perhaps best represented by New Labour in the UK, which substantially expanded UK welfare spending by reaping the tax revenues from a liberalized, financialized economy. The record of countries that have never had very strong labour unions (e.g. the USA or Spain) or that have largely broken their labour movements (e.g. the UK) is not particularly encouraging with regards to inequality. All three are among the countries with the highest Gini coefficients in the OECD.

There are some possible alternatives to organized labour as an agent of equality. France, e.g. shows that relatively healthy public policies can exist in a country with a weak and fragmented union movement if the government explicitly uses spending through the welfare state to compensate for the damage that liberal labour markets can do to welfare.^21^ Few countries, though, have economic policy debates that resemble those of France. Moreover, it is unclear that the French trajectory can or will be sustained. Outside France, Europe has many examples of industrial relations, social protection and workplace regulations that can bias economic outcomes towards public health.

Nonetheless, the direction of travel in *every* wealthy country since the 1980s has been towards a more decentralized labour market with greater employer discretion.^22^ In some cases there are also clear policy decisions to damage unions that are not obvious to all observers, such as the decision of right wing Nordic governments to remove unions from their role in the unemployment insurance system.^23^ That predictably lowered union membership and decreased union power, which in turn makes future erosion of union power and wage egalitarianism easier. Labour market systems in most EU countries have been resilient, but often for reasons that are not clearly beneficial. If industrial relations and workplace regulation systems increasingly depress wages to compete in the Eurozone, or to maintain entire classes of bureaucratic intermediaries,^24^ then their benefits will not look so impressive to voters.

The result of this liberal shift has been increasing income inequality and precarity across the wealthy countries. Persisting differences between, e.g. Austria and the UK, should not obscure this trend. The trend towards greater employer discretion and inequality between workers should lead to a series of negative public health effects as each age cohort suffers the consequences. It is hard to imagine that any set of politically plausible public programmes could compensate for spiraling inequality in globalized liberal labour markets. Public health will thus face a choice: either continue to focus on smaller-scale interventions that partially compensate for the negative public health effects of destructured labour markets, or start to appreciate that industrial relations and workplace regulation are both enormously important drivers of public health outcomes, and inevitably contested political outcomes.
